# Comparative analyses of fungicide sensitivity and SSR marker variations indicate a low risk of developing azoxystrobin resistance in *Phytophthora infestans*

**DOI:** 10.1038/srep20483

**Published:** 2016-02-08

**Authors:** Chun-Fang Qin, Meng-Han He, Feng-Ping Chen, Wen Zhu, Li-Na Yang, E-Jiao Wu, Zheng-Liang Guo, Li-Ping Shang, Jiasui Zhan

**Affiliations:** 1 Fujian Key Laboratory of Plant Virology, Institute of Plant Virology, Fujian Agriculture and Forestry University, Fuzhou, 350002, China; 2 Key Lab for Biopesticide and Chemical Biology, Ministry of Education, Fujian Agriculture and Forestry University, Fuzhou, Fujian, P. R. China

## Abstract

Knowledge of the evolution of fungicide resistance is important in securing sustainable disease management in agricultural systems. In this study, we analyzed and compared the spatial distribution of genetic variation in azoxystrobin sensitivity and SSR markers in 140 *Phytophthora infestans* isolates sampled from seven geographic locations in China. Sensitivity to azoxystrobin and its genetic variation in the pathogen populations was measured by the relative growth rate (RGR) at four fungicide concentrations and determination of the effective concentration for 50% inhibition (EC_50_). We found that all isolates in the current study were sensitive to azoxystrobin and their EC_50_ was similar to that detected from a European population about 20 years ago, suggesting the risk of developing azoxystrobin resistance in *P. infestans* populations is low. Further analyses indicate that reduced genetic variation and high fitness cost in resistant mutations are the likely causes for the low evolutionary likelihood of developing azoxystrobin resistance in the pathogen. We also found a negative correlation between azoxystrobin tolerance in *P. infestans* populations and the mean annual temperature of collection sites, suggesting that global warming may increase the efficiency of using the fungicide to control the late blight.

Synthetic fungicides have played an important role in securing food production, improving social economics and human health^1^,^2^ by increasing crop yields, creating jobs and minimizing the intake of toxic substances produced by pathogens. It is estimated that more than one third of field crop output in the USA can be attributed to the use of agricultural chemicals including fungicides (CropLife America 2011). With the continuing increase in the global human population in the next decades, crop protection with synthetic fungicides is expected to increase.

Synthetic fungicides inhibit or reduce disease development in plants by damaging pathogen cell membranes, inactivating critical enzymes or proteins required for growth and reproduction, interfering with key life processes such as energy production, affecting metabolic pathways such as the formation of sterols and chitin, or by triggering immunity responses in host plants^2^. One of the main problems associated with the intensive use of synthetic fungicides over large areas is the potential for partial or total loss of their efficacy due to the emergence of genotypes in pathogen populations that have the ability to overcome the activity of fungicides. Pathogens achieve fungicide resistances through mutations in target sites, over-expression of target proteins, altering biosynthesis pathways, increased efflux and reduced influx of active ingredients, or changed cell-wall composition^3^,^4^,^5^. Over the last decades, numerous fungicide resistances have occurred world-wide, leading to loss of efficiency in several entire fungicide classes^6^. For example, metalaxyl was used with high efficacy in the late 1970s to combat mildews and potato late blight. Resistance to the fungicide was first documented in *P. infestans* in 1980^7^ and since then has been observed in many pathogens including *Pseudoperonospora cubensis, Plasmopara halstedii* and *Phytophthora erythosepitica*^8^,^9^.

Knowledge of the evolution of fungicide resistance in pathogen populations is important for planning strategies to increase the effective use of fungicides and reduce the costs of plant disease management. Important factors affecting the emergence of fungicide resistance include the mode of action in fungicide itself and the biology and evolutionary ecology of pathogens such as their mating systems, dispersal mechanisms, the genetic variation and interaction with other species^10^,^11^. In addition, human activities, agricultural practices and local environmental factors such as temperature may also contribute to the evolutionary trajectory and emergence of fungicide resistance^12^. Temperature is one of the most important environmental parameters with crucial impacts on all biotic and abiotic processes^13^,^14^,^15^. It can affect the evolutionary landscape of pathogens^16^,^17^, toxicity of chemicals^18^,^19^ and interactions between pathogens and chemicals. While sustainable efforts have been taken to decipher molecular mechanisms of fungicide resistance, field-based empirical studies to understand the evolutionary processes involved in the resistance development are limited. A step towards evolutionary understanding of the resistance can be achieved through a joint population genetic analysis of spatial distributions in fungicide sensitivity and neutral molecular markers^20^,^21^.

Azoxystrobin (Syngenta) is a fungicide which has been commonly used around the world to protect field crops, fruits and vegetables. It is the leading synthetic fungicide in the strobilurin family. The fungicide inhibits mitochondrial respiration of pathogens by binding its active compound to Qo in the cytochrome *bc1* enzyme complex (Complex III), thereby blocking electron transfer and halting ATP synthesis^22^. It was launched to control plant pathogens in 1996^22^ but pathogens with azoxystrobin resistance emerged shortly after. In 1998, resistance to azoxystrobin was first observed in a field population of the wheat powdery mildew pathogen *Blumeria graminis* collected from northern Germany^23^. Since then, field resistance to azoxystrobin has been reported in a range of important plant pathogens worldwide^24^.

Potato late blight caused by *Phytophthora infestans* (Mont) de Bary is among the most important plant diseases in the world. It is infamous as the under-lying cause of the Irish potato famine of the 1840s but still is the most devastating disease globally^25^, particularly in regions of moderate temperature and high humidity. Under favorable climatic conditions, an entire potato crop can be destroyed by the disease within a few days. The pathogen can attack all parts of potato crop including leaves, stems and tubers, resulting in ~6.7 billion US dollars of annually economic losses in the world-wide^26^,^27^.

Potato late blight is mainly controlled by repeated applications of synthetic fungicides including azoxystrobin, together with the deployment of host resistances. In some developed countries, a successful potato harvest may rely on 10–15 fungicide applications each season. However due to its high evolutionary potential, development of resistance in *P. infestans* has been widely documented in phenylamide fungicides and is also a concern of potato growers and agricultural chemical manufacturers for other types of fungicides such as azoxystrobin. Numerous transposable elements distributed around functional genes in the *P. infestans* genome^28^,^29^ may allow the pathogen to generate large amounts of genetic variation to cope with frequent changes in fungicide initiatives; and an epidemic mode of population development^30^ adopted by the pathogen could ensure its ability to preserve allelic combinations that are well adapted to existing fungicides while retaining the pathogen’s ability to generate novel allelic combinations that may offer an evolutionary advantage to counter the introduction of new fungicides^31^.

The objectives of this study were to: i) monitor the spatial distribution of azoxystrobin sensitivity in field populations of *P. infestans* occurring in the main Chinese potato cropping regions; ii) investigate the relative role of genetics and plasticity in determining azoxystrobin resistance; and iii) infer the main evolutionary forces driving the evolution of azoxystrobin resistance in *P. infestans* by comparing spatial distributions of genetic variation in SSR marker loci and azoxystrobin sensitivity.

## Results

### Frequency distribution of azoxystrobin sensitivity in field populations of *P. infestans*

**Figure 1:**
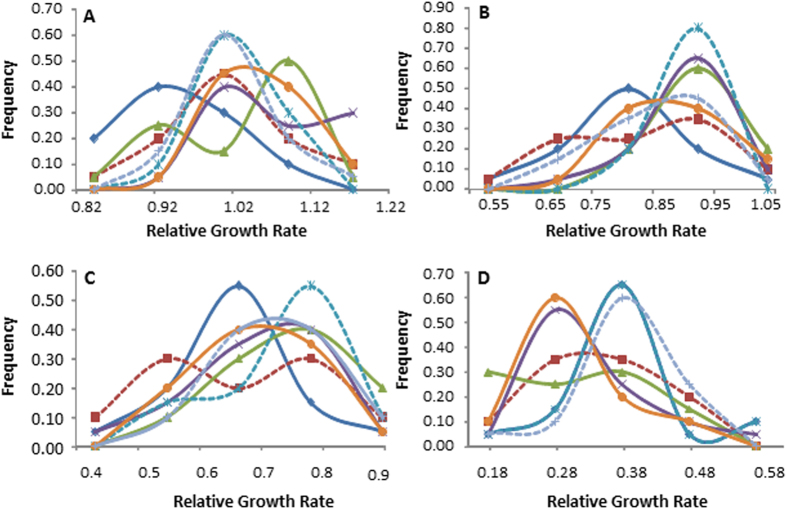
Frequency distribution of azoxystrobin tolerance in the individual field (Fuzhou 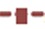, Gansu 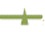, Guangxi 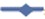, Guizhou 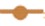, Ningxia 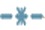, Xiapu 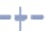 and Yannan 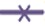) populations of *P. infestans* measured by relative growth rate (RGR) in the presence and absence of the fungicide: (**A**) 0.01 μg/ml azoxystrobin; (**B**) 0.05 μg/ml azoxystrobin: (**C**) 0.10 μg/ml azoxystrobin and (**D**) 0.15 μg/ml azoxystrobin.

**Figure 2:**
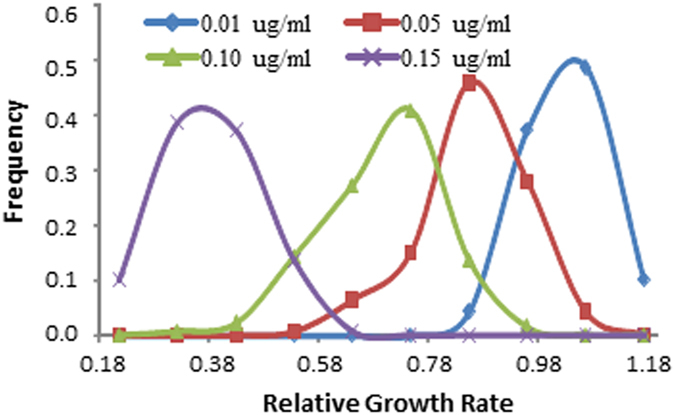
Frequency distribution of azoxystrobin tolerance measured by relative growth rate (RGR) in the presence and absence of the fungicide in the *P. infestans* population combined from different locations.

Twenty each of clonal lineages (140 total) sampled from seven locations across China were tested for sensitivity to four concentrations of azoxystrobin by calculating the relative growth rate (RGR) of the pathogen in the presence and absence of the fungicide. In all four azoxystrobin treatments, RGR displayed a continuous and unimodal distribution in all but one (Fuzhou) field populations of *P. infestans* (Fig. 1). More than 45% of the *P. infestans* isolates grew better on the medium supplemented with 0.01 μg/ml of azoxystrobin than on the medium lacking any fungicide. When the concentration of azoxystrobin in the medium increased to 0.05 μg/ml, only one of the 140 isolates grew better on the medium with than without azoxystrobin supplementations. Growth was inhibited in all isolates when the concentration of azoxystrobin in the medium reached either 0.10 or 0.15 μg/ml (Fig. 1). RGR also displayed a continuous and unimodal distribution in all four azoxystrobin treatments when isolates from different locations were pooled (Fig. 2) and the ratio of RGR in the fastest and slowest growth isolates increased when the concentration of azoxystrobin increased. At the 0.01 μg/ml azoxystrobin treatment, the ratio of RGR between the fastest and slowest growth isolates was 1.56 but this value increased to 2.21 at 0.05 μg/ml treatment, 3.85 at 0.10 μg/ml treatment and 5.56 at 0.15 μg/ml treatments.

**Figure 3:**
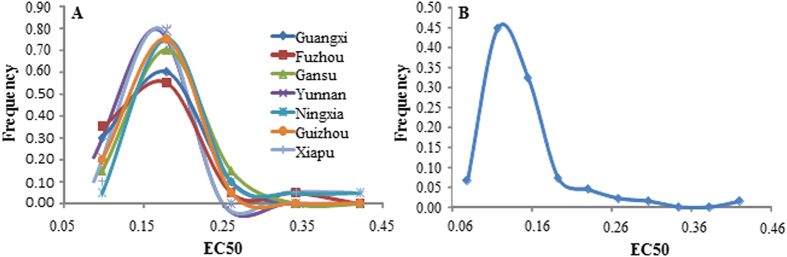
Frequency distribution of azoxystrobin tolerance in *P. infestans* measured by effective concentration for 50% inhibition (EC_50_): (**A**) individual field populations sampled from different locations; and (**B**) Pooled population by combining the isolates from different locations together.

Effective concentration for 50% inhibition (EC_50_) was calculated for each isolate using its RGR in four azoxystrobin concentrations. Though peaking at different levels, EC_50_ also displayed a continuous and unimodal distribution in both individual field (Fig. 3A) and pooled (Fig. 3B) populations, with a range from 0.042 μg/ml in the most sensitive isolate to 0.26 μg/ml in the least sensitive isolate when isolates from different locations were considered together.

### Genetic variation in SSR marker loci and azoxystrobin tolerance

Molecular variation of the pathogen was estimated using eight SSR marker loci. The average SSR diversity in the seven field *P. infestans* populations ranged from 0.39 to 0.49 with a SSR diversity of 0.50 when the isolates from the seven populations were pooled (Table 1). The *P. infestans* population collected from Xiapu displayed the highest gene diversity while that collected from Ningxia displayed the lowest gene diversity.

The contribution of genetic architecture and gene expression to azoxystrobin sensitivity in the pathogen population was measured by heritability and phenotypic plasticity using a Common Garden approach. Heritability in the seven populations ranged from 0.07 to 0.43 with an average of 0.21 while the plasticity in the seven populations ranged from 0.15 to 0.42 with an average of 0.31 for RGR. The pathogen population collected from Fuzhou displayed the highest heritability while the pathogen population sampled from Gansu displayed the highest plasticity. The ratio of heritability to plasticity in RGR in the seven field populations ranged from 0.26 to 1.16 with an overall ratio of 0.68 when all isolates were pooled. For EC_50_, heritability in the seven field populations ranged from 0.17 to 0.87 with an overall heritability of 0.54 in the pooled population. The pathogen population collected from Fuzhou displayed the highest heritability in EC_50_ while that from Yunnan showed the lowest heritability.

### Differences among *P. infestans* populations in azoxystrobin sensitivity

**Figure 4:**
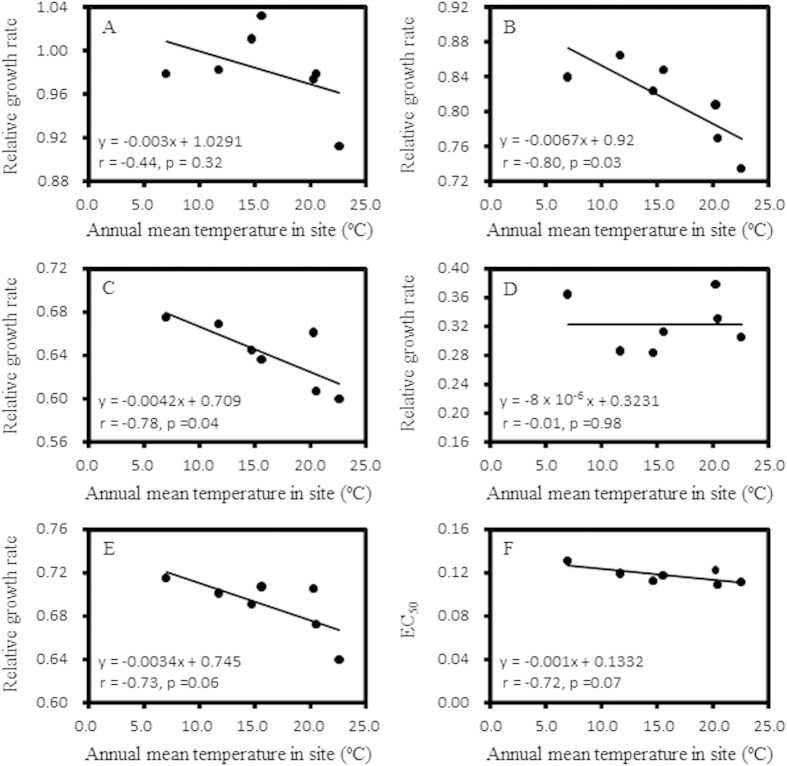
Correlation between annual mean temperature at collection sites and azoxystrobin tolerance of the pathogen (as measured by relative growth rate (RGR) in the presence and absence of the fungicide and effective concentration for 50% inhibition (EC_50_). (**A**) RGR in 0.01 μg/ml azoxystrobin; (**B**) RGR in 0.05 μg/ml azoxystrobin: (**C**) RGR in 0.10 μg/ml azoxystrobin; (**D**) RGR in 0.15 μg/ml azoxystrobin: (**E**) mean RGR over the four azoxystrobin concentrations; and (**F**) EC_50_.

Least significant difference analysis indicated significant differences in RGR and EC_50_ among *P. infestans* isolates sampled from the same or different fields (Tables 2 and 3). The *P. infestans* isolates also responded differently to the dose effect of azoxystrobin (significant isolate x concentration interaction, Table 2). The pathogen population from Ningxia displayed the highest average RGR and EC_50_ while that from Guangxi showed the lowest values (Table 3). Overall, there were negative correlations between both RGR and EC_50_ and the mean annual temperature at sampling locations (Fig. 4).

Population differentiation (*Q*_*ST*_) in azoxystrobin sensitivity was estimated in a way similar to the population differentiation in SSR marker loci (*F*_*ST*_) by calculating the proportion of total quantitative genetic variation attributable to among population variation. The overall population differentiations in RGR, EC_50_ and SSR across the seven pathogen populations were 0.078, 0.072 and 0.116, respectively. The overall *Q*_*ST*_ in RGR and EC_50_ was significantly lower than *F*_*ST*_ for SSR marker loci. The correlation between *F*_*ST*_ in SSR marker loci and *Q*_*ST*_ in RGR and EC_50_ were 0.16 and 0.11, respectively, but none were significant.

## Discussion

In agriculture, increasing attention has been paid to understand and manage the development of field resistance to synthetic fungicides in plant pathogens. To mitigate the risk of loss of efficacy in synthetic fungicides, knowledge of the genetic and evolutionary mechanisms responsible for the development of fungicide resistance in pathogen populations is needed. This can be inferred from statistical analyses of spatial population genetic dynamics. In this study, we investigated the evolutionary development of resistance to azoxystrobin, a leading member of strobilurin fungicides, by comparing the spatial distribution of quantitative genetic variance in azoxystrobin sensitivity with that of molecular variation determined from an SSR assay. Though it has commonly been believed that resistance to strobilurin fungicides is controlled by a single major gene^32^, we found that all our populations displayed a continuous and unimodal distribution in azoxystrobin sensitivity both in terms of RGR and EC_50_ (Figs 1, 2, 3), suggesting other genetic or physiological mechanisms may also be involved in the evolution of azoxystrobin resistance in *P. infestans*.

Mutants with reduced sensitivity have been observed in laboratories and fields in many plant pathogens^24^. However, our analysis indicates that no such resistance has developed in the field populations of *P. infestans* in China. In this study, only seven field populations with a total of 140 isolates were tested for azoxystrobin resistance. The common garden approach we adopted allowed us to estimate genetic variances associated with azoxystrobin ressitance without conducting sexual crosses between isolates but requires all experimental units to be treated under the same conditions within the same time period ideally by the same person^21^,^31^, limiting the number of isolates/populations that can be included in the study. Thougn constrained by number of populations assayed, our results are consistent with previous studies conducted in other continents, The EC_50_ in our study is slightly higher than the value detected in Serbian populations (0.02 to 0.07 μg/ml)^33^ but within the range detected in Swiss ones (0.04 to 3.00 μg/ml)^34^. The Swiss populations were assayed in 1996 at the time when azoxystrobin was first introduced into commercial use and can be considered as the baseline sensitivity of *P. infestans* to the fungicide. Since its introduction, azoxystrobin has been widely used to control plant pathogens included *P. infestans* in the geographic areas covered by the current study. No major change in sensitivity profile between the studies conducted ~20 years apart suggests that the risk of developing azoxystrobin resistance in the field populations of *P. infestans* may be low.

One important factor affecting the development of fungicide resistance in pathogen populations is genetic variation. Fisher’s fundamental theorem of natural selection states that the ability of species to adapt to changing environments depends on their additive genetic variance in ecological and morphological characters that are relevant to fitness^35^. This theory suggests that the risk of developing fungicide resistance is lower in pathogen pathogens with low genetic variation than ones with high genetic variation. Apparently, genetic variation for azoxystrobin resistance in *P. infestans* populations is low. Variance analysis indicates that most of the variation in azoxystrobin tolerance is caused by environmental error and plasticity (Table 1). On average, genetic variance accounted for less than a quarter of the phenotypic variation. In some populations such as Guizhou and Ningxia, genetic variation contributes to less than 10% of the total variation.

Azoxystrobin resistance in pathogens is usually caused by mutation of the cytochrome b gene in the mitochondrial genome. Low genetic variation for azoxystrobin resistance in *P. infestans* populations may be due to slower evolutionary rates in its mitochondrial genome relative to its nuclear genome. It has been documented that many fungi, oomycetes and plants have a lower base substitution rate in the mitochondrial genome than in the nuclear genome^36^ and *P. infestans* may display a similar pattern of mitochondrial evolution. In addition to mutation, recombination is another evolutionary force generating genetic variation within species. Pathogen populations with regular recombination are expected to display high genetic variation either through reshuffling of existing alleles or the creating new alleles. Unlike its nuclear genome, inheritance in mitochondria in *P. infestans* is uniparental^37^.

Natural selection may also explain the low genetic variation of azoxystrobin sensitivity found in *P. infestans*. In addition to the fungicide targeted site, mitochondrial genomes contain many other genes that are vital for the survival and reproduction of *P. infestans.* Due to uniparental inheritance^37^, natural selection is expected to be very effective in purging genetic variation of mitochondrial genes. Indeed, this is consistent with our comparative analysis of spatial distribution in genetic variation showing that population differentiation in azoxystrobin sensitivity is significantly lower than that in SSR neutral markers, therefore suggesting constrained selection for the quantitative trait.

Constraining evolution occurs when environments in different locations select for (or against) the same characters^38^. In the evolution of fungicide resistance, selection for resistant mutants due to their ability to reduce the efficacy of fungicides when the fungicides are used over wide geographic locations or selection against resistant mutants due to severe fitness penalties, can lead to constraining evolution. However, we believe the constraining evolution observed in our study is likely to be caused by fitness costs associated with mutations to azoxystrobin resistance because sensitivity to the strobilurin fungicide in *P. infestans* populations has not changed since it was introduced into agriculture 20 years ago^34^.

We found that some *P. infestans* isolates (>45%) grew better on the agar supplemented with than without azoxystrobin, under low fungicide doses (0.01 μg/ml). A similar scenario of increasing pathogen growth under low fungicide doses has been observed in many other pathogen-fungicide interactions^21^. Though we do not know its genetic or physiological mechanisms, this observation suggests that low doses of azoxystrobin or other fungicides with similar action modes may actually promote the growth of pathogens. This possibility should be taken into account when field applications are contemplated.

It is interesting to find a negative association between fungicide tolerance and local temperature (Fig. 4), suggesting that, on average, *P. infestans* populations from warmer locations are more sensitive to azoxystrobin than those from cooler locations. Temperature is one of main factors regulating the chemical features of molecular compounds. Numerous studies have shown that mortality in animals increases when they are treated with pesticides under elevated temperatures^18^,^19^. In those studies, mortality was usually assessed under different temperature schemes using the same set of animal genotypes and pesticides. It is not clear whether the mortality increase is due to an increased toxicity of the pesticides, an increased animal sensitivity or an interaction between the animal and the pesticide. In our study, we conducted the experiment under constant temperature and we therefore believe that the observed difference in the sensitivity of *P. infestans* may not be due to toxicity changes in the fungicide.

It is projected that average temperatures may increase a few of degrees in the next decades^39^. Such a trend in global air temperature may intensify plant disease occurrence and severity in agriculture^40^,^41^. The finding of a negative correlation between azoxystrobin resistance in *P. infestans* and mean annual temperature suggests that global warming may increase the sensitivity of *P. infestans* to the synthetic fungicide, thereby increasing fungicide efficiency and reducing its applications needed. Though our results suggest that the risk of developing azoxystrobin resistance in *P. infestans* populations is low, further study with the combination of an experimental evolution approach and molecular analysis of target genes is required to confirm the conclusion.

## Materials and Methods

### *Phytophthora infestans* collection and isolation

**Figure 5:**
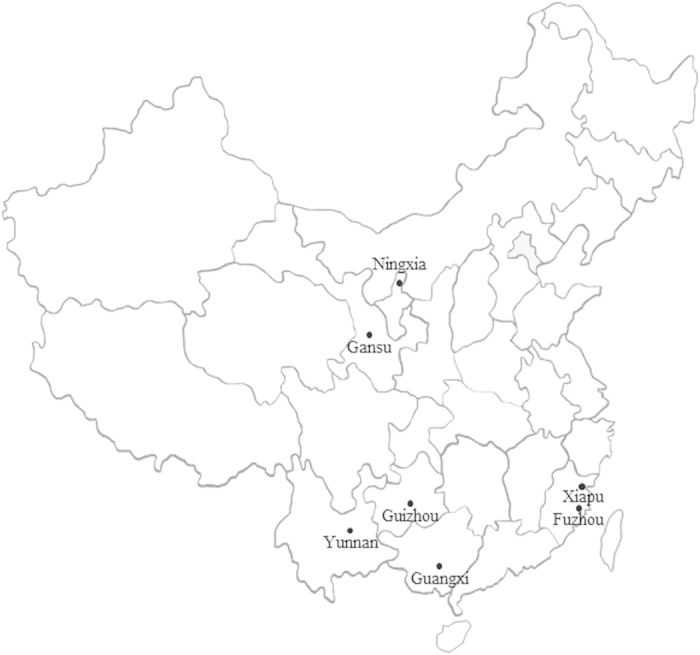
Map showing the geographic locations of the seven *Phytophthora infestans* populations included in this study. Adobe Illustrator Artwork 17.0 software (https://2acd-downloads.phpnuke.org/en/c62216/adobe-illustrator) was used to create the map.

Potato leaves infected with *P. infestans* were sampled from seven fields located in Fuzhou, Gansu, Guangxi, Guizhou, Ningxia, Xiapu and Yunnan during the 2010 and 2011 growing seasons (Fig. 5). Gansu, Guizhou, Ningxia and Yunnan are among the top potato production areas in China while Guangxi and Fujian (Fuzhou and Xiapu), both located in Winter Cropping region, are the two provinces with the highest potential of developing potato industry in next decades attributable to governmental promotion and change of dietary structure in China. For all collections, infected leaves were sampled at random from plants separated by 1–2 meters and transported to the laboratory within 24 hours for isolation. To isolate the pathogen, infected leaves were first rinsed with running water for 60 seconds and then with sterilized distilled water for 30 seconds. A piece of tissue was cut from the margin of a leaf lesion and placed abaxial side up on 2.0% water agar for 20–30 hours. A single piece of mycelium was removed aseptically from the sporulating lesion using an inoculating needle, transferred to a rye B agar plate supplemented with ampicillin (100 μg/ml) and rifampin (10 μg/ml) and maintained at 18 °C in the dark for seven days to develop colony. Purification was performed by twosequential transfers of a single piece of mycelium hyphae tipped from the colony to a fresh rye B plate. The resulted isolate was maintained in long-term storage until further use.

### DNA extraction

Mycelia (~100 mg) were obtained by culturing *P. infestans* isolates on rye B agar at 18 °C in the dark for 15 days, transferred into sterile, 2 mL centrifuge tubes and lyophilized with a vacuum freeze dryer (Alpha1-2, Christ, Germany). The lyophilized mycelia were ground to a powder with a mixer mill (MM400, Retsch, Germany). Total DNA was extracted using a Plant gDNA Miniprep Kit (GD 2611, Biomiga, China) according to the manufacturer’s instructions. The genomic DNA was suspended in 200 μL of ultrapure water and stored at −20 °C.

### SSR analysis

Genomic DNA from each of the *P. infestans* isolates was amplified with eight pairs of SSR primers^42^,^43^ labeled with fluorescent dyes^30^. PCR amplification was performed in a 25 μL volume in a micro tube containing 1.0 μL of *P. infestans* genomic DNA (~20 ng), 12.5 μL of 2× PCR Buffer Mix (TransGen Biotech Co., Ltd., Beijing, China), 1.0 μM each of forward and reverse primers in a 2720 thermal cycler (Applied Bionsystems, USA) with the following conditions: initiated with a cycle of 2 min at 94 °C, followed by 35 cycles of 30 s at 94 °C, 25 s at 56–58 °C (dependent on the primers) and 60 s at 72 °C, and finished with an elongation cycle of 5 min at 72 °C. PCR products were loaded into 96-well plates and sent to Ruiboxingke Biotechn. Co. Ltd. (Beijing) to determine fragment sizes using an ABI 3730XL automated DNA sequencer (Applied Biosystems, Foster, California) in which a DNA size ladder was included in each of the samples^30^. Alleles were assigned using GeneMarker software version 3.7 with a binning procedure.

### Experimental test for azoxystrobin sensitivity

A total of 140 clonal lineages (20 from each of the seven *P. infestans* populations) were selected to test for azoxystrobin sensitivity according to a Common Garden design^38^,^44^,^45^. *P. infestans* isolates from long-term storage were revived on rye B agar at 18 °C for 10 days. Mycelia plugs (3 mm in diameter) were taken from the margin of each revived colony and inoculated onto new rye B plates supplemented either with (treatments) or without (controls) azoxystrobin (Sigma, Aldrich). Azoxystrobin concentrations used in the experiment were 0.01, 0.05, 0.10 and 0.15 μg/ml. Preliminary experiments indicate these doses yielded the best result in differentiating azoxystrobin sensitivity among strains. Many isolates did not grow when a higher dose was used while growth rates in many isolates were not significantly changed when a lower dose was used. The azoxystrobin was first dissolved in dimethyl sulfoxide to make a stock solution and then diluted with double distilled water to the required concentrations. Inoculated plates were kept in the dark at 18 °C and resultant colonies were photographed 3, 5, 7, 8 and 9 days after inoculation. Colony sizes were measured with the image analysis software Assess. All treatments including controls had three replicates.

### Data Analyses

Growth rates of *P. infestans* isolates in azoxystrobin treatments and controls were estimated using an exponential model^46^ based on the sizes of individual colonies quantified at each time point over the experiment. Azoxystrobin sensitivity of *P. infestans* was estimated from the relative growth rate (RGR) and effective concentration for 50% inhibition (EC_50_). RGR of isolates were calculated by dividing the growth rate of an isolate in the presence of azoxystrobin with that in the absence of the fungicide. EC_50_ was calculated as described previously^47^, using the inhibition rates of each isolate in four azoxystrobin concentrations.

Gene diversity^48^ and genetic differentiation in the SSR loci were estimated using Popgene 3.2^49^. Phenotypic variance for RGR was partitioned into sources attributable to isolate (I, random effect), population (P, random effect) and fungicide concentration (C, fixed effect) using SAS GLM and VARCOMP programs (SAS 9.4, SAS Institute) according to the model:


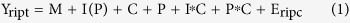


where Y_ripc_, M, P, I(P), I*C,P*C *E*_*ript*_ refer to the mean RGR of replicate *r* for isolate *i* in population *p* at concentration *c*, the overall population mean, genetic variance among populations, genetic variance within populations, variance due to genotype x concentration interaction, responses of populations to dose effect and the variance among replicates, respectively. In common garden experiments with asexually reproducing species, any among-replicate variation in the phenotypic value of an isolate can be treated as environmental effect. Therefore, variance among replicates in this case is equivalent to the environmental variance of RGR^38^,^44^,^45^.

For EC_50_, the model is reduced to:


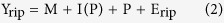


where Y_rip_ is the mean EC_50_ of replicate *r* for isolate *i* in population *p.* M, I(P), P and E_rip_ indicate the overall mean, genetic variance within populations, genetic variance among populations and variance between replicates, respectively.

Heritability was estimated by dividing genetic variance within populations with total phenotypic variance^50^ and plasticity was calculated by dividing the variance of isolate x concentration interaction with total phenotypic variance^51^. The standard deviations for heritability and plasticity were generated from 100 bootstraps of the original data.

Population differentiation in RGR was estimated with following formula^38^,^45^,^52^:


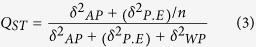


where δ^2^_AP_, δ^2^_WP_, δ^2^_PC_, δ^2^_P·E_ and *n* are among population variance, within population variance, the variance in population x concentration interaction and the number of environments (concentrations), respectively. For EC_50_, Q_ST_ was calculated using the following formula^38^,^45^:


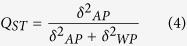


where δ^2^_AP_ is the genetic variance in EC_50_ attributed to among population variation and δ^2^_WP_ is the genetic variance in EC_50_ attributed to within population variation. Like *F*_*ST*_*, Q*_*ST*_ for RGR and EC_50_ was also calculated for all possible pairs of populations as well as across all populations.

Statistical differences between the overall *F*_*ST*_ in SSR loci and overall *Q*_*ST*_ in azoxystrobin sensitivity was evaluated using the standard deviation of *Q*_*ST*_ constructed from 100 resampling of original data as described previously^45^. Least significant difference^53^ was used to compare RGR and EC_50_ among *P. infestans* populations sampled from different locations. Temperature data for each collection site was downloaded from World Climate (http://www.worldclimate.com/). Annual temperature at each location was estimated based on the mean temperature for each month. Pearson correlation^54^ was used to evaluate the association between azoxystrobin sensitivity and the mean annual temperature at the sampling location.

## Additional Information

**How to cite this article**: Qin, C.-F. *et al.* Comparative analyses of fungicide sensitivity and SSR marker variations indicate a low risk of developing azoxystrobin resistance in *Phytophthora infestans. Sci. Rep.* **6**, 20483; doi: 10.1038/srep20483 (2016).

## Figures and Tables

**Table 1:** Variations in SSR marker loci and azoxystrobin tolerance in field populations of *P. infestans*.

Population	Isolate	RGR			SSR diversity	EC_50_Heritability
		**Heritability (H)**	**Plasticity (P)**	**H:P**		
Fuzhou	20	0.43	0.37	1.16	0.44	0.87
Gansu	20	0.10	0.42	0.24	0.46	0.55
Guangxi	20	0.37	0.34	1.09	0.43	0.76
Guizhou	20	0.07	0.27	0.26	0.40	0.24
Ningxia	20	0.09	0.15	0.60	0.39	0.36
Xiapu	20	0.23	0.21	1.10	0.49	0.58
Yunnan	20	0.17	0.39	0.44	0.47	0.17
Total	140	0.21	0.31	0.68	0.50	0.50

**Table 2:** Analysis of variance for azoxystrobin tolerance among the *P. infestans* populations sampled from seven locations in China.

Parameter	Source	D. F	F value	P
RGR	Population	6	26.38	<0.0001
	Isolates	133	8.53	<0.0001
	Concentration	3	6159.18	<0.0001
	Isolate*concentration	417	3.42	<0.0001
	Error	1679	6.31	<0.0001
EC_50_	Population	6	39.3	<0.0001
	Isolates	133	6.31	<0.0001
	Error	264	39.3	<0.0001

**Table 3:** The relative growth rate (RGR) in the presence and absence of azoxystrobin and effective concentration for 50% inhibition (EC_50_) in seven *Phytophthora infestans* populations.

Population	RGR					EC_50_	
	0.01 μg/ml	0.05 μg/ml,	0.10 μg/ml,	0.15 μg/ml,	Mean	Mean	Highest : lowest
Fuzhou	0.979B	0.770AB	0.607A	0.331ABC	0.672AB	0.109A	3.39
Gansu	0.983B	0.865C	0.669C	0.286A	0.705C	0.119AB	1.76
Guangxi	0.912A	0.735A	0.600A	0.305AB	0.640A	0.111A	3.18
Guizhou	1.011BC	0.824BC	0.645ABC	0.284A	0.691C	0.112AB	1.58
Ningxia	0.979B	0.840C	0.675BC	0.364BC	0.715C	0.131C	2.85
Xiapu	0.974B	0.808BC	0.661BC	0.378C	0.706C	0.122BC	2.17
Yunnan	1.032C	0.848C	0.636AB	0.313AB	0.706C	0.117AB	2.08

Values followed by different letters in the same column differ significantly at P = 0.05.
